# Interpretation of Genomic Variants Using a Unified Biological Network Approach

**DOI:** 10.1371/journal.pcbi.1002886

**Published:** 2013-03-07

**Authors:** Ekta Khurana, Yao Fu, Jieming Chen, Mark Gerstein

**Affiliations:** 1 Program in Computational Biology and Bioinformatics, Yale University, New Haven, Connecticut, United States of America; 2 Molecular Biophysics and Biochemistry Department, Yale University, New Haven, Connecticut, United States of America; 3 Integrated Graduate Program in Physical and Engineering Biology, Yale University, New Haven, Connecticut, United States of America; 4 Department of Computer Science, Yale University, New Haven, Connecticut, United States of America; University of Chicago, United States of America

## Abstract

The decreasing cost of sequencing is leading to a growing repertoire of personal genomes. However, we are lagging behind in understanding the functional consequences of the millions of variants obtained from sequencing. Global system-wide effects of variants in coding genes are particularly poorly understood. It is known that while variants in some genes can lead to diseases, complete disruption of other genes, called ‘loss-of-function tolerant’, is possible with no obvious effect. Here, we build a systems-based classifier to quantitatively estimate the global perturbation caused by deleterious mutations in each gene. We first survey the degree to which gene centrality in various individual networks and a unified ‘Multinet’ correlates with the tolerance to loss-of-function mutations and evolutionary conservation. We find that functionally significant and highly conserved genes tend to be more central in physical protein-protein and regulatory networks. However, this is not the case for metabolic pathways, where the highly central genes have more duplicated copies and are more tolerant to loss-of-function mutations. Integration of three-dimensional protein structures reveals that the correlation with centrality in the protein-protein interaction network is also seen in terms of the number of interaction interfaces used. Finally, combining all the network and evolutionary properties allows us to build a classifier distinguishing functionally essential and loss-of-function tolerant genes with higher accuracy (AUC = 0.91) than any individual property. Application of the classifier to the whole genome shows its strong potential for interpretation of variants involved in Mendelian diseases and in complex disorders probed by genome-wide association studies.

## Author Summary

The number of personal genomes sequenced has grown rapidly over the last few years and is likely to grow further. In order to use the DNA sequence variants amongst individuals for personalized medicine, we need to understand the functional impact of these variants. Deleterious variants in genes can have a wide spectrum of global effects, ranging from fatal for essential genes to no obvious damaging effect for loss-of-function tolerant genes. The global effect of a gene mutation is largely governed by the diverse biological networks in which the gene participates. Since genes participate in many networks, no singular network captures the global picture of gene interactions. Here we integrate the diverse modes of gene interactions (regulatory, genetic, phosphorylation, signaling, metabolic and physical protein-protein interactions) to create a unified biological network. We then exploit the unique properties of loss-of-function tolerant and essential genes in this unified network to build a computational model that can predict global perturbation caused by deleterious mutations in all genes. Our model can distinguish between these two gene sets with high accuracy and we further show that it can be used for interpretation of variants involved in Mendelian diseases and in complex disorders probed by genome-wide association studies.

## Introduction

Advances in next-generation sequencing technologies have considerably reduced the cost of genome sequencing. As a result, there has been an avalanche of personal genomic data with numerous individual genomes sequenced in the last few years [1]–[4]. Variants in protein-coding genes are of special interest due to their stronger likelihood of functional effects. A comprehensive understanding of the functional impact of variants in coding genes requires their integration with various levels of annotations, such as primary sequence of the gene, three-dimensional structures of its protein products and biological networks where genes interact with each other. Functional annotation of single nucleotide variants (SNVs) at genomic sequence level results in their classification as nonsynonymous (which includes missense and nonsense), splice site disrupting or synonymous. Similarly, small insertions and deletions (indels) in coding genes can be classified as frame-shift or in-frame. Nonsense and splice site disrupting SNVs as well as frame-shift indels are mostly assumed to lead to loss-of-function (LoF) of genes [5]. On the other hand, missense SNVs and in-frame indels may or may not be damaging [6].

It is well understood that genes and their protein products rarely act in isolation but rather work closely with other genes and/or their products to form various networks and pathways which accomplish specific goals, for example, signal transduction, metabolism etc. Thus, a comprehensive understanding of the functional impact of variants necessitates the inclusion of these interactions between genes. Network-based approaches are thus often used to study human disease [7]. One feature that has emerged from past studies of disease genes and networks is that protein products of genes associated with similar disorders have a higher likelihood of physical interaction with each other [8]. It has also been noted in many studies that functionally essential genes are more likely to encode for hub (i.e. highly connected) proteins in the physical protein-protein interaction (PPI) network in both yeast [9] and humans [8]. Moreover, hub proteins are likely to be under stronger negative selection constraints in humans and positive selection tends to occur on network periphery [10]. Similar studies on signaling pathways have revealed that as one goes from extracellular space to the nucleus in the cell, negative selection constraints on genes encoding corresponding proteins tend to increase [11]. Selection studies have also been performed on metabolic pathways where enzyme connectivity signifies the number of other metabolic enzymes that produce the enzyme's reactants or consume its products. For example, in a yeast network of 584 metabolites and comprising about 16% of all yeast genes, Vitkup et al found that highly connected enzymes evolve slower than less connected enzymes [12]. Montanucci et al also reported that genes encoding highly connected enzymes in N-glycosylation metabolic pathway exhibit stronger purifying selection constraints and tend to evolve slowly in primates [13].

In order to obtain a higher resolution understanding of the relationship between selection constraints and networks, some studies have also integrated three-dimensional protein structures with PPI network to obtain structural interaction network (SIN). Kim et al showed that in yeast, hubs in PPI with more than two interaction interfaces are more likely to be essential than those with two or less interfaces [14]. Using structurally resolved human PPI network, Wang et al showed that disease-causing missense SNVs and in-frame insertions and deletions tend to be enriched at the interaction interfaces of proteins associated with corresponding disorders [15]. They also showed that the disease specificity of different mutations of the same gene can be explained by their location on the interaction interfaces. Another important feature that has emerged from studies of genomic variants on protein structure (without consideration of network interactions) is that benign missense polymorphisms tend to occur at solvent exposed sites on protein structure, while disease-causing missense SNVs tend to be more buried [16].

Previous studies examining the relationship of functional significance and selection properties of genes with network topology have mostly focused on networks with a singular mode of interactions between genes or their protein products, for example physical protein-protein interactions. However, a gene and its protein products can be involved in various biological networks and its role and consequently its centrality can vary across these networks. For example, SIX5 is a transcription factor gene that targets 360 genes in the human regulatory network but interacts with only one protein in the physical PPI network [17], [18]. This gene is of high functional significance since its disruption causes Branchio-oto-renal syndrome, a developmental disorder characterized by the association of branchial arch defects, hearing loss and renal anomalies [19]. In this study we examine the relationship of functional essentiality and selection with various biological networks – protein-protein interaction (PPI), phosphorylation, signaling, metabolic, genetic and regulatory. This enables us to understand the functional importance and selection constraints on genes in a global systemic approach. Moreover, although it has been shown that low evolutionary conservation of LoF- tolerant genes and their large distance from recessive disease genes in PPI network can be used to predict disease causation of variants [5], their unique properties in diverse biological networks have not been exploited before. Here, we use the distinguishing network and evolutionary properties of functionally essential and LoF-tolerant genes to build a predictive model for global damage caused by novel variants. Using this model, we are able to compute functional indispensability scores for all protein-coding genes.

## Results

### Building the Multinet

The biological networks studied in this work include – PPI, phosphorylation, metabolic, signaling, genetic and regulatory (Materials and Methods). Some of these networks represent direct physical interactions between proteins, for example, PPI. On the other hand, genetic and regulatory networks contain indirect interactions between gene pairs. Additionally, some networks such as phosphorylation, metabolic, signaling and regulatory are directional with an upstream and downstream gene, whereas PPI and genetic interactions are undirected. While a gene can have a vital role in one pathway or network, it might not be as crucial in another network. Therefore, we pool together data from all the above-mentioned biological networks to construct a unified global network, which we term Multinet (Materials and Methods). The Multinet enables the analyses of the genes via their roles in the individual networks and the combined network.

We note that some interactions between two different networks can be shared. For example, an interaction in which gene A phosphorylates gene B can occur in both phosphorylation and PPI networks. However, we find that out of ∼110,000 interactions in our data set, only 881 interactions occur in more than one network. Thus the vast majority of interactions in our data are unique or non-redundant. This observation reiterates the fact that interactions of genes vary across different networks and it is crucial to include all the networks while analyzing the relationship between functional importance and selection constraints with global network centrality. The distribution of 881 interactions which occur in more than one network is shown in Supporting Figure S1. The numbers of genes and unique interactions in each network are shown in Supporting Table S1.

### Functional essentiality and network properties of genes

In this section we investigate the relationship between functional significance of genes and their properties in various biological networks. All human protein-coding genes are divided into four categories based on their known disease susceptibilities and functional impact. A ‘gene significance score’ ranging from 3 to 0 is assigned to each gene: 3 for essential genes, 2 for all genes with disease-causing mutations in HGMD, 0 for LoF-tolerant genes and 1 for all the remaining genes that do not fit into any of the above categories (Materials and Methods). We then correlate these significance scores with the degree centralities of the genes in all networks. Degree centrality of a gene in any network is defined as the number of its interacting partners in that network. In order to estimate the total number of interacting partners of a gene, we use its connectivity (number of interactions) in the Multinet (Materials and Methods).

**Figure 1:**
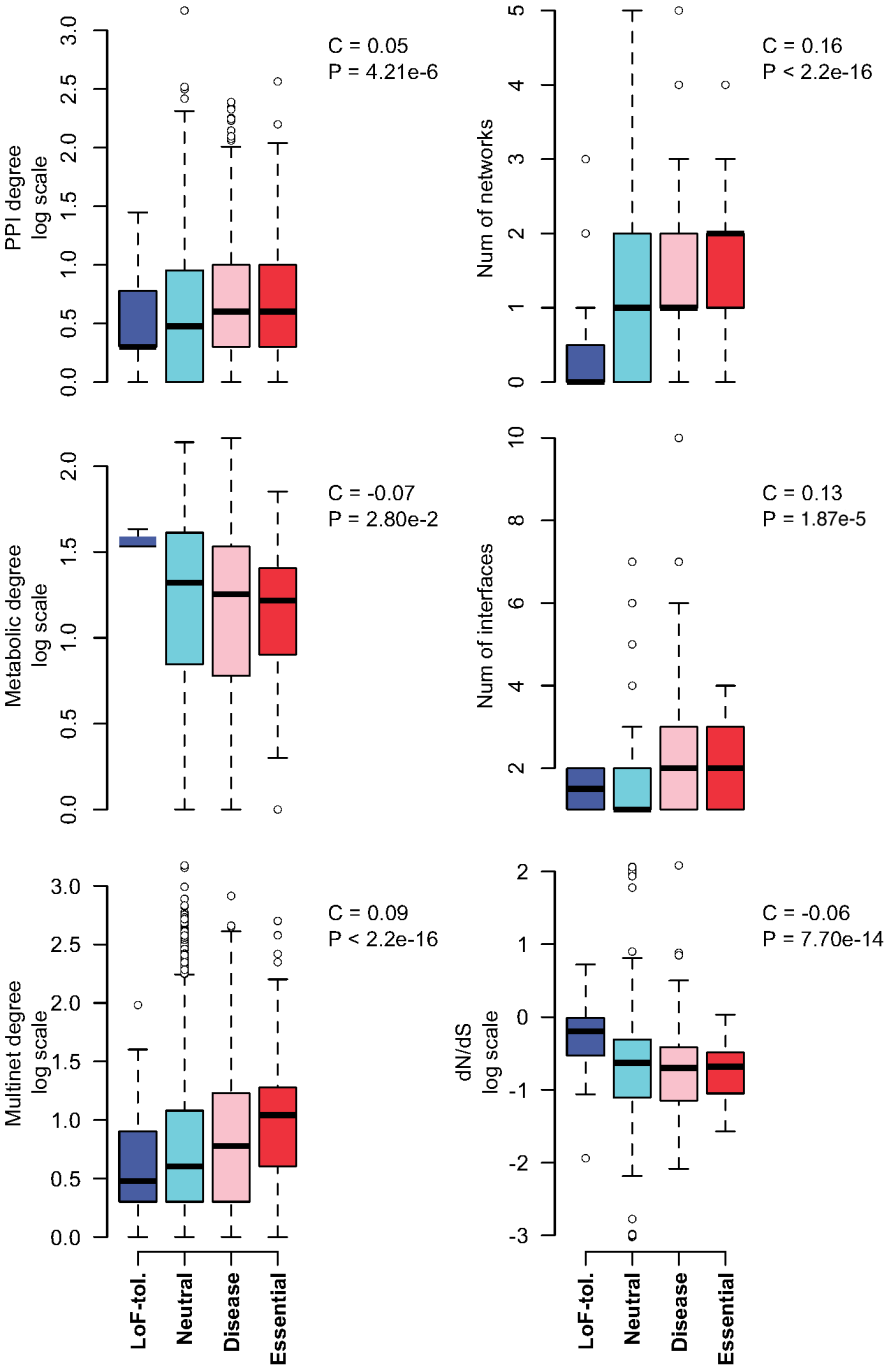
Distributions of various network and evolutionary properties for the four gene categories: LoF-tolerant (blue); neutral (cyan); with known disease-causing mutations (pink) and essential (red). Spearman correlation coefficients (C) between the corresponding property values of all genes with the gene significance scores are shown at the top right of each boxplot. Corresponding pvalues (P) are also shown.

**Figure 2:**
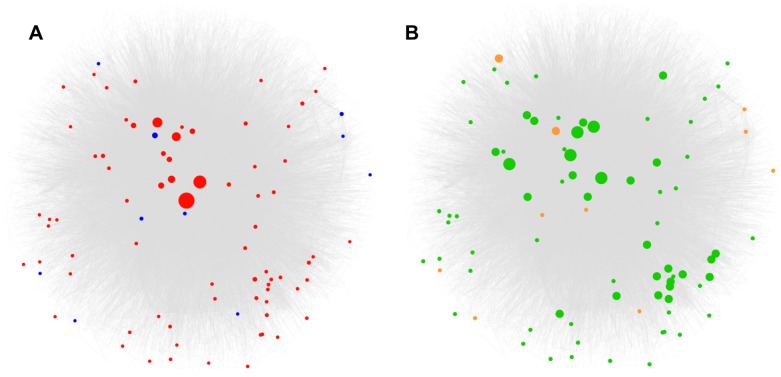
This figure shows the edges (grey) of the Multinet, which correspond to interactions between genes. Only the interactions of genes that are involved in more than one network are shown for clarity. (A) Nodes corresponding to LoF-tolerant and essential genes are shown in blue and red respectively. Size of the nodes is scaled based on the degree centrality of the gene in Multinet. Essential genes tend to be bigger and in the center while LoF-tolerant genes tend to be smaller and on the periphery. (B) Nodes corresponding to LoF-tolerant and essential genes are shown in orange and green respectively. Size of the nodes is scaled based on the number of networks the gene is involved in. Essential genes tend to be involved in more networks and hence are bigger while LoF-tolerant genes are smaller. Most LoF-tolerant genes are not involved in any network and are not present in the Multinet.

We find that gene significance scores show positive correlation with degree centralities in most networks, though it is statistically significant only in PPI and signaling network and Multinet (Figures 1 and 2A; Supporting Table S2). Thus, in general, essential genes tend to be more connected in biological systems consistent with previous findings [8]. Surprisingly, we find a small but significant negative correlation between gene significance score and metabolic degree (Spearman correlation coefficient or SCC = −0.07, pvalue = 0.028). We also find that, unlike most other degree centralities, the metabolic degree centrality of genes shows a significant positive correlation with the number of paralogs (duplicated copies) (SCC = 0.15; pvalue = 8.26e-07) (Supporting Table S3; Materials and Methods). Thus, it is possible that in case of a LoF mutation in a participating enzyme, the metabolic pathway can be re-routed to an alternate path, possibly involving a duplicated gene of the disabled enzyme. Our observation in the human metabolic network is in agreement with a previous study by Vitkup et al, in which they found that highly connected enzymes are no more likely to be essential than less connected enzymes in yeast metabolic network [12]. In this study we find that not only are essential genes unlikely to be highly connected in human metabolic network, LoF-tolerant genes (whenever present in metabolic network) are indeed more connected than essential genes (Supporting Table S7). This result demonstrates a major contrast between the structure of the metabolic network and other networks. In most biological networks, highly connected genes tend to have fewer duplicated copies; hence LoF mutations in them can have serious phenotypic consequences. Since this distinct trend of high degeneracy at hub proteins is observed only in the metabolic network, we further posit that this might be an evolutionary mechanism to increase tolerance towards damaging mutations. The uniqueness of such a ‘protective’ effect somewhat suggests an implicit level of greater functional importance of metabolic pathways as compared to other networks of gene interactions.

Interestingly, we find that gene significance scores are positively correlated with the number of networks the gene is involved in (Figures 1 and 2B). This indicates that genes involved in many networks can act as information bottlenecks between different systems and thus tend to be more essential.

### Selection constraints and network properties of genes

We next examine the relationship between selection constraints on genes and their network properties. We estimate evolutionary constraints over long time-scale by dN/dS (ratio of missense to synonymous substitution rates) computed from human-chimp ortholog alignments (Materials and Methods). dN/dS<1 indicates purifying selection while values close to 1 indicate neutral selection and dN/dS>1 indicates positive selection. We find that dN/dS values of genes are negatively correlated with their degree centralities in all networks, though they reach significance in PPI, phosphorylation, regulatory and Multinet networks (Supporting Table S4). This shows that highly connected genes tend to be under stronger purifying selection constraints over long evolutionary time-scale, in agreement with previous studies [10].

Furthermore, we analyze patterns of genetic variation in modern-day humans in relation to biological networks. We compute average heterozygosity of each gene to estimate its genetic variability using missense SNPs (single nucleotide polymorphisms) and their corresponding allele frequencies in three sets of populations from 1000 Genomes Pilot data (Materials and Methods) [4]. We find that there is a significant negative correlation between Multinet degree and heterozygosity of missense SNPs for all three populations, indicating more genetic variation at the periphery of networks (the correlation is also significant for some populations in PPI, phosphorylation and regulatory networks) (Supporting Table S5). Interestingly, we do not find a significant correlation of heterozygosity of synonymous SNPs with Multinet degree (Supporting Table S6; Materials and Methods). Putting together, these results suggest that reduced genetic variability of highly connected genes with respect to missense SNPs is indeed due to selection constraints.

### Molecular level insights from Structural Interaction Network

**Figure 3:**
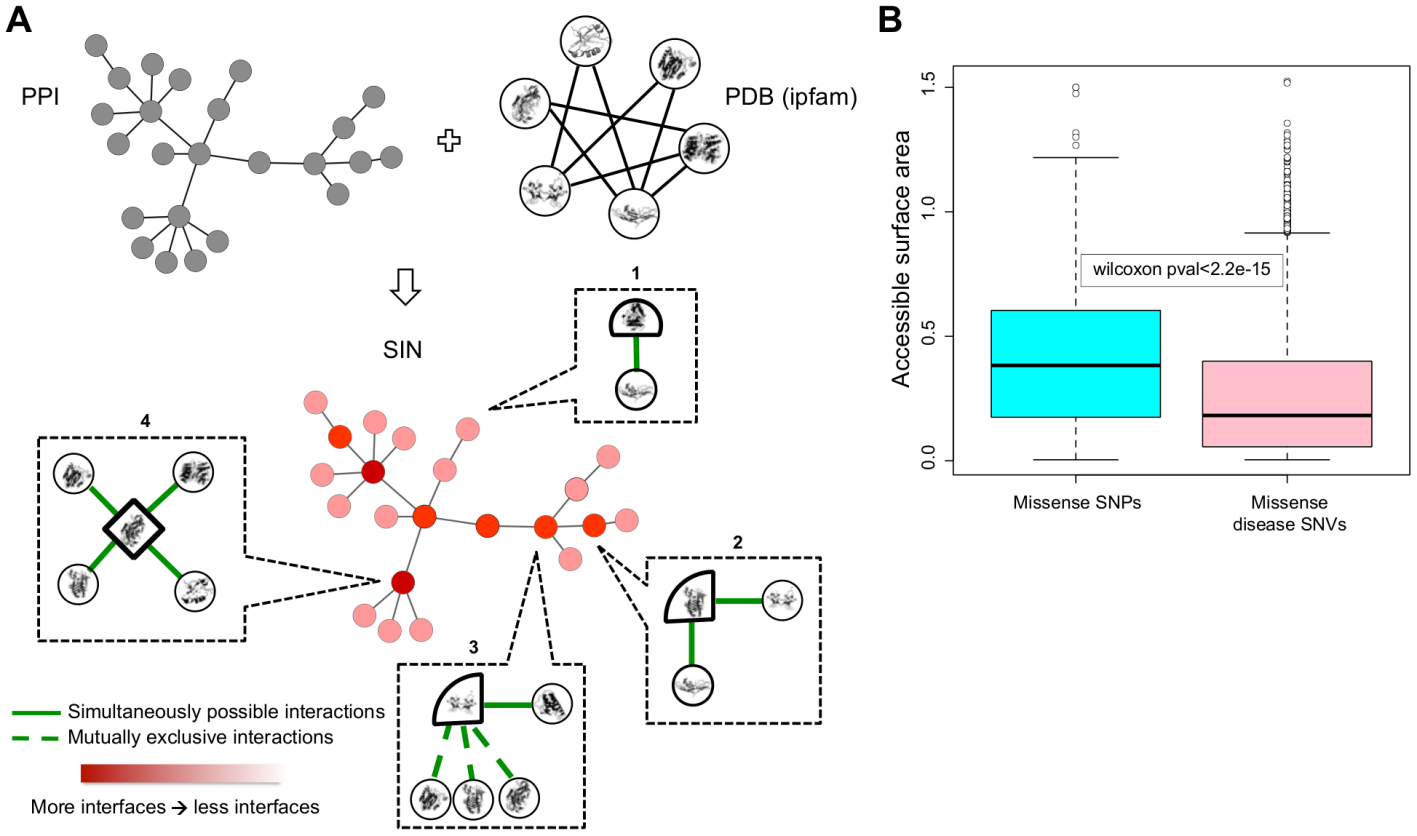
Structural Interaction Network (SIN). (A) Schematic of SIN construction shown by combining three-dimensional protein structural information from ipfam with PPI network. Nodes in resulting SIN are shown in red with color intensity indicating the number of interaction interfaces used by the protein. Closer look is provided for four nodes in SIN. While the surface of proteins is generally shown by circular lines, an interface is depicted using a straight line. Rectangle 1 shows a protein using one interaction interface to interact with one protein; rectangle 2 shows a protein with two simultaneously possible interactions and consequently two interaction interfaces; rectangle 3 shows a protein with three mutually exclusive and one simultaneously possible interactions resulting in two interaction interfaces; rectangle 4 shows four simultaneously possible interactions resulting in four interfaces. (B) Distributions of accessible surface areas of missense SNPs (cyan) and disease-causing missense SNVs (pink).

When network edges between two genes correspond to physical interactions between their protein products, molecular level details of the interaction can be obtained by integrating three-dimensional protein structures with the underlying network data. Therefore, in order to understand the reasons for selection constraints in PPI network at higher resolution, we integrated three-dimensional protein structures with network interaction data to create structural interaction network (SIN) (Figure 3A; Materials and Methods) [14], [15], [20]. SIN is a subset of the larger PPI network and consists of 2,102 genes and 11,433 interactions.

SIN construction allows us to estimate the number of interfaces used by a protein to interact with other proteins (Figure 3A; Materials and Methods). We find that there is a significant positive correlation between gene significance scores and the number of interfaces used by their protein products in SIN (Figure 1). Thus, protein products of essential genes tend to use more interaction interfaces than those of LoF-tolerant genes. We also find that the number of interfaces used by the protein to interact with other proteins in SIN is positively correlated with their degree centrality in PPI network (SCC = 0.18, pvalue = 1.06e-09). This shows that hub proteins tend to have more interaction interfaces. Thus, it is likely that higher number of interfaces possessed by protein products of essential genes could partly be a result of their higher degree centrality in PPI network.

We next examine the impact of missense SNVs on protein structure in relation to SIN. We find that, in general, residues with disease-causing missense SNVs tend to be more buried inside protein structure than polymorphic residues (Figure 3B). Our observation is consistent with previous findings which have reported that missense mutations buried inside protein structure tend to be more deleterious than those on surface [16]. However, these previous studies treated all proteins equally and did not differentiate between hub and non-hub proteins in PPI network. When we treat hub (degree centrality> = 50) and non-hub proteins separately, we find that accessible surface area for residues with missense disease mutations is higher for hub proteins (Wilcoxon rank sum pvalue = 0.014; Supporting Figure S2). We also observe a significant positive correlation between the degree centrality of protein and the accessible surface area of their residues undergoing disease mutations (SCC = 0.028, pvalue = 3.12e-03). These results show that hub proteins tend to have a higher fraction of missense disease mutations on their exposed surface. This result is very reasonable in light of our observation that hub proteins tend to have more interaction interfaces (see preceding paragraph), thereby having a higher fraction of their exposed surface under selection constraints.

### Predicting probable functional indispensability of genes using selection and network properties

**Figure 4:**
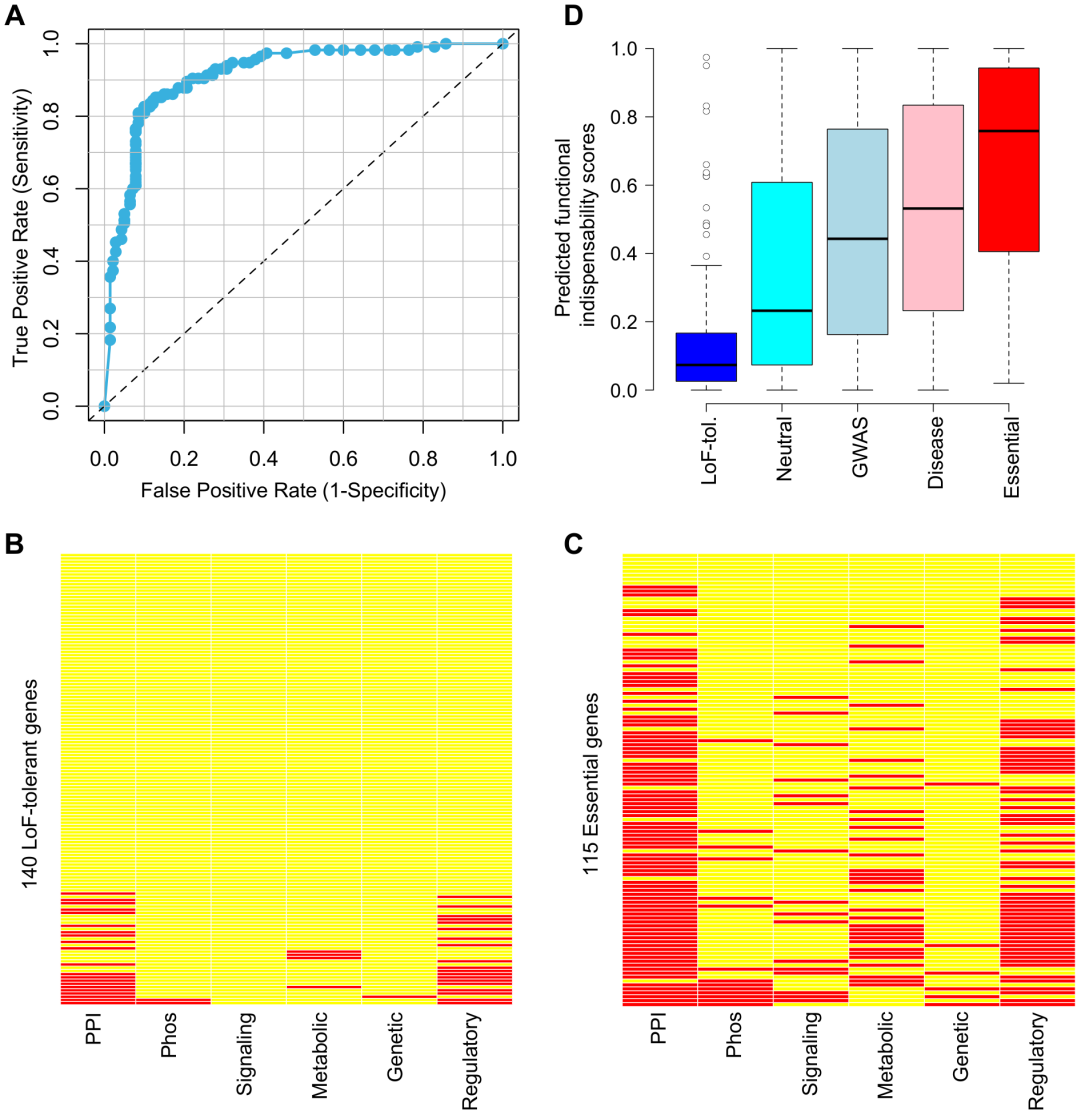
Prediction of functional indispensability scores. (A) ROC curve resulting from cross-validation of the logistic regression model to distinguish LoF-tolerant and essential genes. Participation of (B) LoF-tolerant and (C) essential genes in various networks. Rows correspond to gene names while columns correspond to networks. Presence of a gene in a network is shown by red while its absence is shown by yellow. (D) Distribution of predicted functional indispensability scores for five gene categories: LoF-tolerant (blue); neutral (cyan); identified in GWA studies (light blue); with known disease-causing mutations (pink) and essential (red).

In order to further examine the close correlation of network and evolutionary properties with gene essentiality we use a logistic regression model to differentiate essential genes from LoF-tolerant genes (Materials and Methods). Network features used to train the logistic regression model include degree centralities in Multinet and all networks separately (PPI, phosphorylation, signaling, metabolic, genetic and regulatory), number of networks the gene is involved in and number of interfaces used in SIN. Selection properties used in the model include human-chimp dN/dS ratios and average heterozygosities of both synonymous and missense SNPs in modern human populations. The average values of these features for LoF-tolerant and essential genes along with corresponding Wilcoxon rank sum pvalues are provided in Supporting Table S7 (see also Figure 1). Using these features we obtain an excellent classification accuracy for 140 LoF-tolerant and 115 essential genes with AUC = 0.914 (Figure 4A; Materials and Methods).

Network properties that contribute significantly to the model include degree centralities in regulatory, genetic and metabolic networks as well as number of networks the gene is involved in (Materials and Methods). On further examination of network participation of LoF-tolerant and essential genes, we find that most LoF-tolerant genes are not involved in any network and some of them are involved in a very small number of networks (Figure 4B). On the other hand, most essential genes are involved in many networks (Figure 4C). Genes involved in a variety of networks serve as information bottlenecks between different systems and hence are more likely to be essential. We note that absence in some networks could partially be due to missing network data in our study and/or a bias in existing databases. Essential genes are more likely to have been the focus of previous research studies, for example PPI studies, and hence more likely to be present in our PPI network. They also tend to have more regulatory interactions and thus are more likely to be present in our regulatory network (which consists of 118 transcription factors and their target genes: the most comprehensive human regulatory network available to our knowledge) [17]. However, the strength of our model lies in its use of many different network properties to minimize the biases resulting from the use of a single network property or data resource. Furthermore, to test the robustness of our model, we computed the AUC for separation of LoF-tolerant and essential genes multiple times – each time randomly removing 10% of the edges from a network and rebuilding the Multinet. After repeating this for all the networks, we find minimal change in the AUC (ranging from 0.914 to 0.912), which shows that our model is quite robust to changing some edges in individual networks.

We next perform an independent validation of our model by applying it on all genes that are neither LoF-tolerant nor essential. Interestingly, we find that predicted functional indispensability scores are in the following order: genes with known disease-causing mutations have significantly higher scores than genes identified in genome-wide association (GWA) studies (Wilcoxon rank sum pvalue = 7.62e-05), which are in turn significantly higher than all the remaining neutral genes (Wilcoxon rank sum pvalue<2.2e-16) (Figure 4D). Genes identified in GWA studies are associated with phenotypic consequences, while they are not necessarily the causal genes. Hence it is reassuring that genes with known disease-causing mutations in HGMD receive significantly higher scores than those identified in GWA studies. This validation exercise demonstrates that our model can help researchers pick candidate disease genes in clinical sequencing studies. We have provided the predicted scores for all the genes in Supporting Table S8. We note that the predicted functional indispensability scores are continuous scores unlike the discrete gene significance scores used to compute correlations in an earlier section.

## Discussion

Genes and their protein products work in collaboration with other genes to form biological systems that perform specific tasks. For a systemic understanding of the role a gene plays, there is a need to integrate different modes of gene interactions. In this work we pool together interaction data from various biological systems (PPI, phosphorylation, signaling, metabolic, genetic and regulatory) to create a unified Multinet, enabling the computation of degree centrality of the genes in their individual networks and in the context of the entire Multinet (Supporting Table S8). Subsequent analysis of functional significance and evolutionary properties of genes allows us to relate genomic sequence variants in individual genes to their functional effects in individual and global networks. We find that highly connected genes in the Multinet and genes that participate in many biological systems tend to be more functionally significant, have fewer paralogs and resist mutations in healthy humans. While we also observe similar trends in most of the constituent networks of the Multinet, the metabolic network seems to be an exception. Highly connected genes in the metabolic network tend to have more paralogs and are more tolerant to LoF mutations.

Next, we combine three-dimensional protein structural information with PPI network to create structural interaction network (SIN) and understand selection on protein structure at molecular level detail. We find that functionally essential genes (which are more likely to encode for hub proteins) tend to use more interfaces to interact with other proteins. We also observe that hub proteins in PPI network contain a higher fraction of disease-causing mutations on their solvent exposed surface, as compared to non-hub proteins. Thus, although generally missense SNVs on exposed protein surface are more likely to be benign, our results show that those on the surface of hub proteins are more likely to be deleterious [21].

Finally, we integrate network and selection properties of genes to build a logistic regression model which can separate LoF-tolerant and essential genes with high accuracy (AUC = 0.91). Application of the model on all genes shows that it predicts higher functional indispensability scores for genes with known disease-causing mutations than genes identified in GWA studies, which themselves have higher scores than remaining neutral genes. The predicted functional indispensability scores for all genes are made publicly available and can be used to predict candidate disease genes in future clinical studies. These scores are indicators of global damage caused by deleterious mutations in coding genes – including nonsense and missense SNVs and in-frame and frame-shift indels. As mentioned above, nonsense SNVs and frame-shift indels are mostly assumed to disable gene function. However, missense SNVs and in-frame indels are more complex since they may or may not have a deleterious impact. Various methods exist to predict the functional effects of missense SNVs, for example, SIFT and PolyPhen [21], [22]. While these methods examine the tolerance of individual sites in genes to missense mutations, they do not take into account the functional significance of the entire gene. For example, a moderately deleterious missense SNV in a highly significant gene can be equally or more damaging than a strongly deleterious missense SNV in a less significant gene. Our method to compute functional indispensability scores for entire genes can be combined with scores predicted by SIFT and PolyPhen to obtain a more comprehensive view of the functional effects of genomic variation.

We note that even though our model is very robust to the removal of some edges in individual networks, the incomplete and biased nature of existing biological networks data may constitute a caveat in our study. However, to our knowledge, this is the first comprehensive genome-wide study linking genetic variants at population scale as well as disease variants with a vast body of available network resources. Models developed and applied in this study can be further expanded as more interaction data is obtained and further population genetics projects are undertaken, particularly with the future phases of the 1000 Genomes project.

## Materials and Methods

### Networks data and building Multinet

Human protein-protein interaction and genetic interaction networks were extracted from BIOGRID (release 3.1.83) [18] containing 43,722 and 263 interactions, respectively. Regulatory network (relationship between transcription factors and target genes) was from ENCODE data [17]. Metabolic enzyme network contained directed linkages from upstream enzymes to downstream enzymes, based on compound reactions in KEGG [23]. Phosphorylation network in human contains 28,637 directed kinase-substrate interactions between 2,392 genes [24]. The signaling network in this study is constructed based on 1,011 interactions and 527 proteins (downloaded July 2011) from human signaling pathways obtained from the SignaLink database (http://www.signalink.org/) [25]. SignaLink offers an easily-downloadable and well-curated set of interactions from eight major signaling pathways found in humans that are not tissue-specific, namely EGF/MAPK, Ins/IGF, TGF-β, Wnt, Hedgehog, JAK/STAT, Notch and NHR (Nuclear Hormone Receptors). Manual data curation was performed in SignaLink by extensive literature survey of primary experimental evidence of these interactions, resulting in expansion of verified interaction data for the corresponding signaling pathways in protein interaction databases such as the KEGG [26], Reactome [27] and NetPath [28], while maintaining substantial overlaps with these databases. A detailed description of the curation process and comparisons between these databases and SignaLink can be found in [25]. Throughout the article, connectivity of the gene in PPI, phosphorylation, signaling and metabolic networks refers to connectivity of the protein product of the gene.

Interactions from all the above networks were combined to create Multinet. If a gene pair interacts in multiple networks or shows both upstream and downstream connection in a directional network, the interaction is counted once in Multinet.

### Gene categories

The list of 140 LoF-tolerant genes was obtained from MacArthur et al [5]. This list contains genes that show homozygous LoF mutations in at least one individual in 1000 Genomes pilot data [4]. The list of 115 essential genes was obtained from Liao et al [29]. These genes exhibit clinical features of death before puberty or infertility when LoF mutations occur. The list of 2,451 disease genes was obtained from HGMD (Human Gene Mutation Database) [30]. All the genes with any disease-causing mutation (DM tag in HGMD) were used. If any gene occurred in more than one category, its category was decided in a hierarchical fashion as follows: essential, followed by disease followed by LoF-tolerant. The remaining 19,267 genes were assigned the category of neutral. The list of genes identified in GWA studies was obtained from the NHGRI GWAS catalogue (https://www.genome.gov/26525384#download).

### Obtaining paralogs and dN/dS values for human-chimp orthologs

Number of paralogs for each gene and dN/dS values for human-chimp orthologs were obtained from Ensembl using BioMart [31].

### Source of 1000 Genomes data and calculation of average heterozygosity

SNPs in modern-day humans and their allele frequencies were obtained from the low-coverage pilot phase of the 1000 Genomes Project [4]. This phase consisted of 60 individuals of CEU (Utah residents with Northern and Western European Ancestry), 59 individuals of YRI (Yoruba in Ibadan, Nigeria) and 60 individuals of CHB+JPT (Han Chinese in Beijing, China and Japanese in Tokyo, Japan) populations. Heterozygosity value is calculated as 2pq, where p and q correspond to the frequencies of the two alleles. Average heterozygosity for a gene is defined as the average heterozygosity of the SNPs in that gene, where heterozygosities of missense and synonymous SNPs are computed separately.

### Structural Interaction Network (SIN)

#### Data

Protein-protein interaction (PPI) network is curated and filtered from HPRD (Human Protein Reference Database) and MIPS database, containing 39,849 interactions between 7,432 proteins [32]. For each protein, the domain information is obtained from Pfam. Pfam domain-domain interactions (DDI) and residue level interactions between protein domains in PDB are obtained from iPfam (release 20.0) [33].

#### SIN construction

Domain-domain interactions are mapped onto protein-protein interaction network through the protein-domain relationships. Interactions that are supported by both DDI and PPI are included in the SIN. Generally speaking, SIN has the interacting domain information in corresponding protein-protein interactions. SIN contains 11,433 domain interactions between 2,262 proteins.

#### Determining interaction types and number of interaction interfaces

Some interactions in SIN are mutually exclusive as they compete for same interaction interface while some of them could occur at the same time, as they use different interfaces (called simultaneously possible interactions). Types of interactions are determined by overlaps in interacting residues. For example, if most residues (>20%) used by protein A to interact with protein B are also used by protein A to interact with protein C, these two interactions of protein A are mutually exclusive and correspond to the same interaction interface. If protein A also interacts with protein D using a completely different set of residues, its interaction with protein D is possible simultaneously to its interactions with protein B or protein C. Thus, for each protein, the interactions it is involved in can be classified into mutually exclusive and simultaneously possible based on the residues used for those interactions. Number of interaction interfaces of a protein is then defined as the number of simultaneously possible interactions involving that protein.

#### Solvent accessible surface area

In order to match the genomic coordinates for 1000 Genomes SNPs and disease-causing SNVs with PDB residue numbers, we first obtained Uniprot/Swissprot accession ids for gene IDs using BioMart [31]. Pairwise alignments of Uniprot sequences and Ensembl sequences gave the matching residue number. Then we found the corresponding PDB id, residue number and its solvent accessible surface area using the data resource provided at http://genetics.bwh.harvard.edu/pph2/dokuwiki/downloads.

### Calculation of functional indispensability scores

#### Logistic regression model

Logistic regression was employed to separate essential genes from LoF-tolerant genes, based on their network and evolutionary properties. We decided to train the model on extremes of gene categories, i.e, LoF-tolerant and essential genes since we have relatively higher confidence in these gene sets. Missing values of a feature for any gene were replaced by the average value of that feature. To improve fitness of model, optimal features were selected using forward feature selection procedure conducted using AIC (Akaike information criterion). This resulted in the addition of the following set of features to the model sequentially: number of networks, dN/dS, regulatory degree, genetic degree, CEU synonymous heterozygosity and metabolic degree.

#### Cross-validation

To evaluate prediction accuracy and minimize over-fitting problems, 10-fold cross-validation was performed and corresponding AUC (area under curve) scores were calculated. AUC scores correspond to areas under the ROC (receiver operating characteristic) curves, which depict the relationship between sensitivity and specificity. The average AUC score from 10-fold cross-validation is reported. Model obtained from training data was then applied to disease genes and non-disease causing (neutral) genes to assess their functional indispensability. A probable functional indispensability score is assigned to each gene, with high score suggesting more likely to be essential.

## Supporting Information

Figure S1 Number of pair-wise gene interactions shared between different networks.
(PDF)

Figure S2 (A) Accessible surface area of sites with missense disease-causing SNVs in hubs is significantly greater than for sites with missense disease-causing SNVs in non-hubs (B) Distribution of gene degree centralities in SIN (structural interaction network).
(PDF)

Table S1 Number of genes and unique interactions in each network.
(PDF)

Table S2 Spearman correlation coefficient (SCC) of gene significance scores with degree centralities in various networks. Pvalues<0.05 denote significant correlations and are shaded in grey.
(PDF)

Table S3 Spearman correlation coefficient (SCC) of number of gene paralogs with degree centralities in various networks. Pvalues<0.05 denote significant correlations and are shaded in grey.
(PDF)

Table S4 Spearman correlation coefficient (SCC) of gene dN/dS values with degree centralities in various networks. Pvalues<0.05 denote significant correlations and are shaded in grey.
(PDF)

Table S5 Spearman correlation coefficient (SCC) of average heterozygosity of missense SNPs for each gene with degree centralities in various networks. Values for each population are shown separately. Pvalues<0.05 denote significant correlations and are shaded in grey.
(PDF)

Table S6 Spearman correlation coefficient (SCC) of average heterozygosity of synonymous SNPs for each gene with degree centralities in various networks. Values for each population are shown separately.
(PDF)

Table S7 Average values of different properties for LoF-tolerant and Essential genes. Wilcoxon rank sum pvalues<0.05 are shaded in grey and denote significantly different distributions of the corresponding property for the two gene categories.
(PDF)

Table S8 Network properties and functional indispensability scores for all genes.
(TXT)

## References

[1] Levy S, Sutton G, Ng PC, Feuk L, Halpern AL, et al (2007) The diploid genome sequence of an individual human. PLoS Biol 5: e254. 17803354 10.1371/journal.pbio.0050254 PMC1964779

[2] Wheeler DA, Srinivasan M, Egholm M, Shen Y, Chen L, et al (2008) The complete genome of an individual by massively parallel DNA sequencing. Nature 452: 872–876. 18421352 10.1038/nature06884

[3] Wang J, Wang W, Li R, Li Y, Tian G, et al (2008) The diploid genome sequence of an Asian individual. Nature 456: 60–65. 18987735 10.1038/nature07484 PMC2716080

[4] Genomes Project Consortium (2010) A map of human genome variation from population-scale sequencing. Nature 467: 1061–1073. 20981092 10.1038/nature09534 PMC3042601

[5] MacArthur DG, Balasubramanian S, Frankish A, Huang N, Morris J, et al (2012) A systematic survey of loss-of-function variants in human protein-coding genes. Science 335: 823–828. 22344438 10.1126/science.1215040 PMC3299548

[6] Ng PC, Henikoff S (2006) Predicting the effects of amino acid substitutions on protein function. Annu Rev Genomics Hum Genet 7: 61–80. 16824020 10.1146/annurev.genom.7.080505.115630

[7] Wang X, Gulbahce N, Yu H (2011) Network-based methods for human disease gene prediction. Brief Funct Genomics 10: 280–293. 21764832 10.1093/bfgp/elr024

[8] Goh KI, Cusick ME, Valle D, Childs B, Vidal M, et al (2007) The human disease network. Proc Natl Acad Sci U S A 104: 8685–8690. 17502601 10.1073/pnas.0701361104 PMC1885563

[9] Jeong H, Mason SP, Barabasi AL, Oltvai ZN (2001) Lethality and centrality in protein networks. Nature 411: 41–42. 11333967 10.1038/35075138

[10] Kim PM, Korbel JO, Gerstein MB (2007) Positive selection at the protein network periphery: evaluation in terms of structural constraints and cellular context. Proc Natl Acad Sci U S A 104: 20274–20279. 18077332 10.1073/pnas.0710183104 PMC2154421

[11] Cui Q, Purisima EO, Wang E (2009) Protein evolution on a human signaling network. BMC Syst Biol 3: 21. 19226461 10.1186/1752-0509-3-21 PMC2649034

[12] Vitkup D, Kharchenko P, Wagner A (2006) Influence of metabolic network structure and function on enzyme evolution. Genome Biol 7: R39. 16684370 10.1186/gb-2006-7-5-r39 PMC1779518

[13] Montanucci L, Laayouni H, Dall'Olio GM, Bertranpetit J (2011) Molecular evolution and network-level analysis of the N-glycosylation metabolic pathway across primates. Mol Biol Evol 28: 813–823. 20924085 10.1093/molbev/msq259

[14] Kim PM, Lu LJ, Xia Y, Gerstein MB (2006) Relating three-dimensional structures to protein networks provides evolutionary insights. Science 314: 1938–1941. 17185604 10.1126/science.1136174

[15] Wang X, Wei X, Thijssen B, Das J, Lipkin SM, et al (2012) Three-dimensional reconstruction of protein networks provides insight into human genetic disease. Nat Biotechnol 30: 159–164. 22252508 10.1038/nbt.2106 PMC3708476

[16] Saunders CT, Baker D (2002) Evaluation of structural and evolutionary contributions to deleterious mutation prediction. J Mol Biol 322: 891–901. 12270722 10.1016/s0022-2836(02)00813-6

[17] Gerstein M, Kundaje A, Hariharan M, Landt S, Yan K, et al (2012) Architecture of the human regulatory network derived from ENCODE data. Nature 489 (7414) 91–100. 22955619 10.1038/nature11245 PMC4154057

[18] Stark C, Breitkreutz BJ, Reguly T, Boucher L, Breitkreutz A, et al (2006) BioGRID: a general repository for interaction datasets. Nucleic Acids Res 34: D535–539. 16381927 10.1093/nar/gkj109 PMC1347471

[19] Hoskins BE, Cramer CH, Silvius D, Zou D, Raymond RM, et al (2007) Transcription factor SIX5 is mutated in patients with branchio-oto-renal syndrome. Am J Hum Genet 80: 800–804. 17357085 10.1086/513322 PMC1852719

[20] Bhardwaj N, Abyzov A, Clarke D, Shou C, Gerstein MB (2011) Integration of protein motions with molecular networks reveals different mechanisms for permanent and transient interactions. Protein Sci 20: 1745–1754. 21826754 10.1002/pro.710 PMC3218368

[21] Adzhubei IA, Schmidt S, Peshkin L, Ramensky VE, Gerasimova A, et al (2010) A method and server for predicting damaging missense mutations. Nat Methods 7: 248–249. 20354512 10.1038/nmeth0410-248 PMC2855889

[22] Kumar P, Henikoff S, Ng PC (2009) Predicting the effects of coding non-synonymous variants on protein function using the SIFT algorithm. Nat Protoc 4: 1073–1081. 19561590 10.1038/nprot.2009.86

[23] Kanehisa M, Goto S, Furumichi M, Tanabe M, Hirakawa M (2010) KEGG for representation and analysis of molecular networks involving diseases and drugs. Nucleic Acids Res 38: D355–360. 19880382 10.1093/nar/gkp896 PMC2808910

[24] Lin J, Xie Z, Zhu H, Qian J (2010) Understanding protein phosphorylation on a systems level. Brief Funct Genomics 9: 32–42. 20056723 10.1093/bfgp/elp045 PMC3096446

[25] Korcsmaros T, Farkas IJ, Szalay MS, Rovo P, Fazekas D, et al (2010) Uniformly curated signaling pathways reveal tissue-specific cross-talks and support drug target discovery. Bioinformatics 26: 2042–2050. 20542890 10.1093/bioinformatics/btq310

[26] Ogata H, Goto S, Sato K, Fujibuchi W, Bono H, et al (1999) KEGG: Kyoto Encyclopedia of Genes and Genomes. Nucleic Acids Res 27: 29–34. 9847135 10.1093/nar/27.1.29 PMC148090

[27] Joshi-Tope G, Gillespie M, Vastrik I, D'Eustachio P, Schmidt E, et al (2005) Reactome: a knowledgebase of biological pathways. Nucleic Acids Res 33: D428–432. 15608231 10.1093/nar/gki072 PMC540026

[28] Kandasamy K, Mohan SS, Raju R, Keerthikumar S, Kumar GS, et al (2010) NetPath: a public resource of curated signal transduction pathways. Genome Biol 11: R3. 20067622 10.1186/gb-2010-11-1-r3 PMC2847715

[29] Liao BY, Zhang J (2008) Null mutations in human and mouse orthologs frequently result in different phenotypes. Proc Natl Acad Sci U S A 105: 6987–6992. 18458337 10.1073/pnas.0800387105 PMC2383943

[30] Stenson PD, Mort M, Ball EV, Howells K, Phillips AD, et al (2009) The Human Gene Mutation Database: 2008 update. Genome Med 1: 13. 19348700 10.1186/gm13 PMC2651586

[31] Flicek P, Amode MR, Barrell D, Beal K, Brent S, et al (2012) Ensembl 2012. Nucleic Acids Res 40: D84–90. 22086963 10.1093/nar/gkr991 PMC3245178

[32] Pagel P, Kovac S, Oesterheld M, Brauner B, Dunger-Kaltenbach I, et al (2005) The MIPS mammalian protein-protein interaction database. Bioinformatics 21: 832–834. 15531608 10.1093/bioinformatics/bti115

[33] Finn RD, Marshall M, Bateman A (2005) iPfam: visualization of protein-protein interactions in PDB at domain and amino acid resolutions. Bioinformatics 21: 410–412. 15353450 10.1093/bioinformatics/bti011

