# Supplementary figures and images for: Interpretation of Genomic Variants Using a Unified Biological Network Approach
Source: PLoS Comput Biol. 2013 Mar 7;9(3):e1002886. doi: 10.1371/journal.pcbi.1002886 (PMC3591262; doi:10.1371/journal.pcbi.1002886)

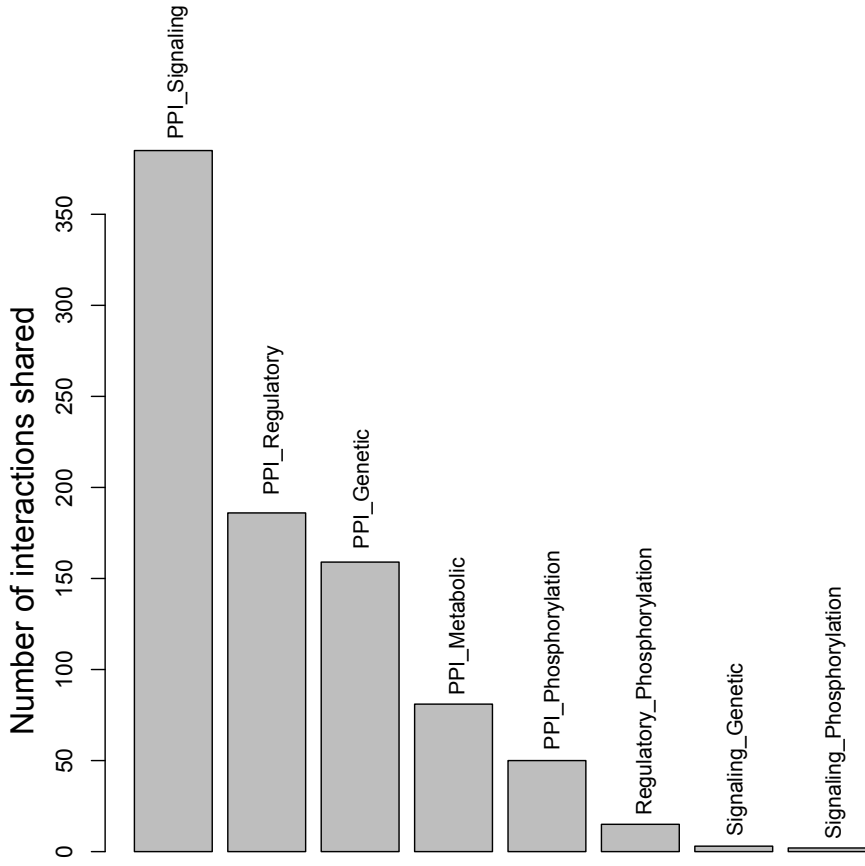

Supplement: Figure S1 — Number of pair-wise gene interactions shared between different networks. (PDF) [file pcbi.1002886.s001.pdf]

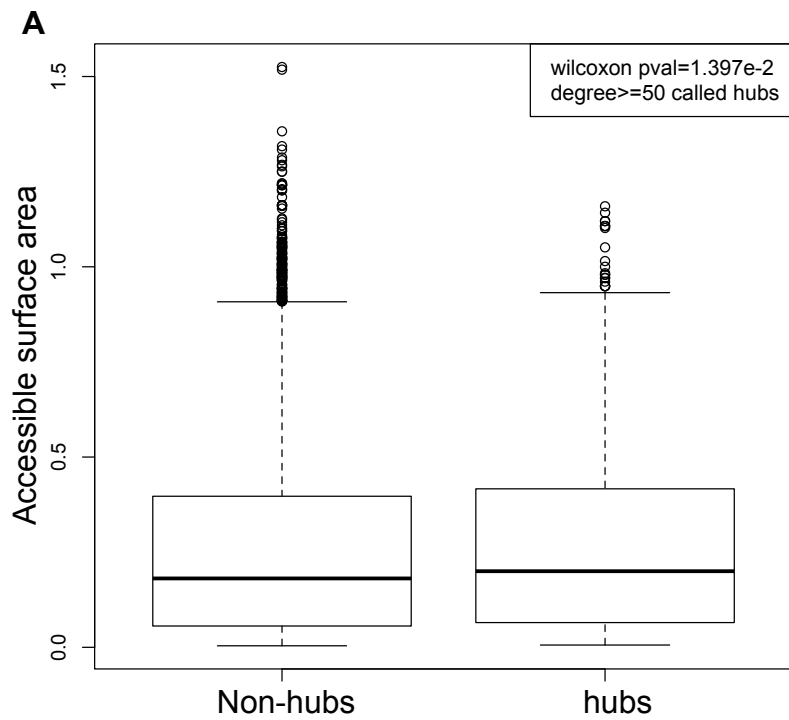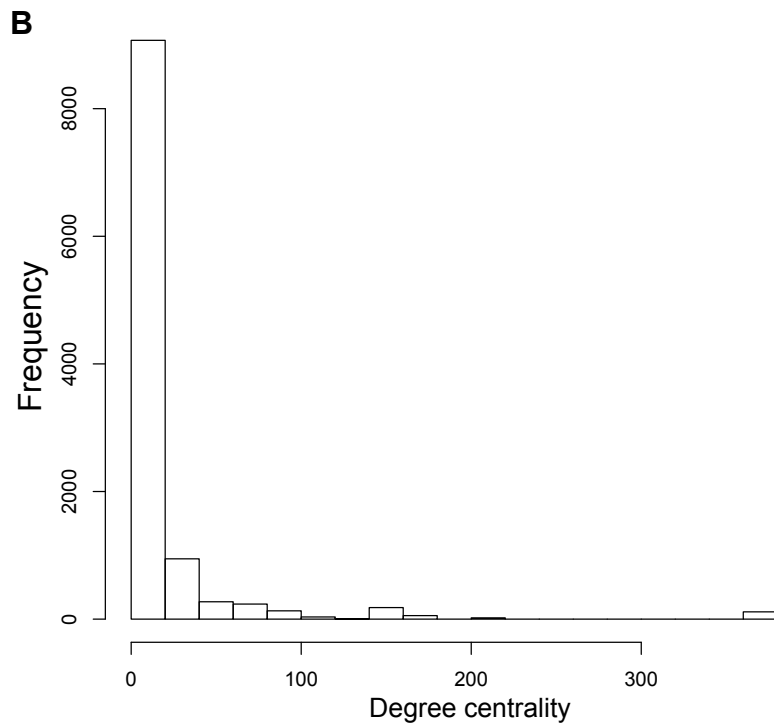

Supplement: Figure S2 — (A) Accessible surface area of sites with missense disease-causing SNVs in hubs is significantly greater than for sites with missense disease-causing SNVs in non-hubs (B) Distribution of gene degree centralities in SIN (structural interaction network). (PDF) [file pcbi.1002886.s002.pdf]
