# Supplementary material for: Interpretation of Genomic Variants Using a Unified Biological Network Approach
Source: PLoS Comput Biol. 2013 Mar 7;9(3):e1002886. doi: 10.1371/journal.pcbi.1002886 (PMC3591262; doi:10.1371/journal.pcbi.1002886)
Supplement: Table S1 — Number of genes and unique interactions in each network. (PDF) [file pcbi.1002886.s003.pdf]

Network	Number of genes	Number of unique interactions
PPI	9,612	43,722
Phosphorylation	2,392	27,900
Signaling	530	994
Metabolic	1,035	9,714
Genetic	272	263
Regulatory	9,314	27,914
Multinet	14,443	109,598
