# Supplementary material for: Interpretation of Genomic Variants Using a Unified Biological Network Approach
Source: PLoS Comput Biol. 2013 Mar 7;9(3):e1002886. doi: 10.1371/journal.pcbi.1002886 (PMC3591262; doi:10.1371/journal.pcbi.1002886)
Supplement: Table S2 — Spearman correlation coefficient (SCC) of gene significance scores with degree centralities in various networks. Pvalues<0.05 denote significant correlations and are shaded in grey. (PDF) [file pcbi.1002886.s004.pdf]

Network	SCC	pvalue
PPI	0.05	4.21e-06
Phosphorylation	-0.01	5.99e-01
Signaling	0.094	3.08e-02
Metabolic	-0.068	2.80e-02
Genetic	0.04	5.13e-01
Regulatory	0.003	7.91e-01
Multinet	0.089	<2.2e-16
