# Supplementary material for: Interpretation of Genomic Variants Using a Unified Biological Network Approach
Source: PLoS Comput Biol. 2013 Mar 7;9(3):e1002886. doi: 10.1371/journal.pcbi.1002886 (PMC3591262; doi:10.1371/journal.pcbi.1002886)
Supplement: Table S3 — Spearman correlation coefficient (SCC) of number of gene paralogs with degree centralities in various networks. Pvalues<0.05 denote significant correlations and are shaded in grey. (PDF) [file pcbi.1002886.s005.pdf]

**Network**	**SCC**	**pvalue**
PPI	-0.0054	6.08e-01
Phosphorylation	0.099	4.85e-06
Signaling	-0.034	4.41e-1
Metabolic	0.15	8.26e-07
Genetic	-0.099	1.02e-01
Regulatory	-0.037	9.06e-04
Multinet	-0.011	2.10e-01
