# Supplementary material for: Interpretation of Genomic Variants Using a Unified Biological Network Approach
Source: PLoS Comput Biol. 2013 Mar 7;9(3):e1002886. doi: 10.1371/journal.pcbi.1002886 (PMC3591262; doi:10.1371/journal.pcbi.1002886)
Supplement: Table S4 — Spearman correlation coefficient (SCC) of gene dN/dS values with degree centralities in various networks. Pvalues<0.05 denote significant correlations and are shaded in grey. (PDF) [file pcbi.1002886.s006.pdf]

**Network**	**SCC**	**pvalue**
PPI	-0.155	<2.2e-16
Signaling	-0.0252	5.789e-1
Phosphorylation	-0.0909	5.46e-5
Metabolic	-0.0167	6.119e-1
Genetic	-0.0333	6.055e-1
Regulatory	-0.0552	1.752e-6
Multinet	-0.156	<2.2e-16
