# Supplementary material for: Interpretation of Genomic Variants Using a Unified Biological Network Approach
Source: PLoS Comput Biol. 2013 Mar 7;9(3):e1002886. doi: 10.1371/journal.pcbi.1002886 (PMC3591262; doi:10.1371/journal.pcbi.1002886)
Supplement: Table S5 — Spearman correlation coefficient (SCC) of average heterozygosity of missense SNPs for each gene with degree centralities in various networks. Values for each population are shown separately. Pvalues<0.05 denote significant correlations and are shaded in grey. (PDF) [file pcbi.1002886.s007.pdf]

Network	SCC (CEU)	Pvalue (CEU)	SCC (YRI)	Pvalue (YRI)	SCC (CHBJPT)	Pvalue (CHBJPT)
PPI	-0.07	7.922e-7	-0.067	8.327e-7	-0.025	9.534e-2
Signaling	-0.071	2.696e-1	0.067	2.806e-1	0.028	6.698e-1
Phosphorylation	-0.063	4.565e-2	-0.0078	7.96e-1	0.0089	7.86e-1
Metabolic	-0.034	4.011e-1	-0.063	1.079e-1	-0.055	2.006e-1
Genetic	0.0047	9.596e-1	-0.002	9.774e-1	0.029	7.688e-1
Regulatory	-0.013	3.634e-1	-0.031	2.461e-2	0.0083	5.886e-1
Multinet	-0.081	6.189e-12	-0.067	2.222e-9	-0.03	1.368e-2
