# Supplementary material for: Interpretation of Genomic Variants Using a Unified Biological Network Approach
Source: PLoS Comput Biol. 2013 Mar 7;9(3):e1002886. doi: 10.1371/journal.pcbi.1002886 (PMC3591262; doi:10.1371/journal.pcbi.1002886)
Supplement: Table S6 — Spearman correlation coefficient (SCC) of average heterozygosity of synonymous SNPs for each gene with degree centralities in various networks. Values for each population are shown separately. (PDF) [file pcbi.1002886.s008.pdf]

Network	SCC (CEU)	Pvalue (CEU)	SCC (YRI)	Pvalue (YRI)	SCC (CHBJPT)	Pvalue (CHBJPT)
PPI	-0.013	2.778e-1	-0.01	3.829e-1	0.0066	5.777e-1
Signaling	-0.057	2.497e-1	0.029	5.548e-1	-0.022	6.606e-1
Phosphorylation	0.019	4.344e-1	0.027	2.724e-1	0.013	6.158e-1
Metabolic	0.051	1.416e-1	0.016	6.479e-1	0.046	1.879e-1
Genetic	0.035	6.128e-1	0.061	3.724e-1	-0.04	5.553e-1
Regulatory	-0.011	3.782e-1	-0.017	1.666e-1	-0.0084	4.966e-1
Multinet	0.0039	6.922e-1	-0.013	1.95e-1	-0.0029	7.722e-1
