# Supplementary material for: Interpretation of Genomic Variants Using a Unified Biological Network Approach
Source: PLoS Comput Biol. 2013 Mar 7;9(3):e1002886. doi: 10.1371/journal.pcbi.1002886 (PMC3591262; doi:10.1371/journal.pcbi.1002886)
Supplement: Table S7 — Average values of different properties for LoF-tolerant and Essential genes. Wilcoxon rank sum pvalues<0.05 are shaded in grey and denote significantly different distributions of the corresponding property for the two gene categories. (PDF) [file pcbi.1002886.s009.pdf]

| Property | Average LoF-tolerant | Average Essential | Wilcoxon pvalue |
|---|---|---|---|
| PPI degree | 6.05 | 15.96 | 8.63e-02 |
| Signaling degree | NA | 2.94 | NA |
| Phosphorylation degree | 42 | 50.64 | 6.84e-01 |
| Metabolic degree | 36.75 | 19.63 | 1.09e-02 |
| Genetic degree | 1 | 3.33 | 2.18e-1 |
| Regulatory degree | 1.84 | 10.02 | 2.80e-02 |
| Multinet degree | 11.09 | 28.95 | 3.53e-04 |
| Number of networks | 0.34 | 1.85 | <2.2e-16 |
| Number of interfaces | 1.5 | 2.2 | 2.64e-01 |
| dN/dS | 0.86 | 0.26 | 1.49e-14 |

| Property (Heterozygosity) | Average LoF-tolerant | Average Essential | Wilcoxon pvalue |
|---|---|---|---|
| CEU missense | 0.23 | 0.18 | 2.09e-03 |
| YRI missense | 0.19 | 0.17 | 1.90e-02 |
| CHBJPT missense | 0.26 | 0.23 | 8.16e-02 |
| CEU synonymous | 0.23 | 0.20 | 2.52e-02 |
| YRI synonymous | 0.20 | 0.19 | 4.73e-01 |
| CHBJPT synonymous | 0.25 | 0.20 | 3.97e-03 |
